# Postoperative resource utilization and survival among liver transplant recipients with Model for End-stage Liver Disease score ≥40: a retrospective cohort study

**DOI:** 10.1186/cc13391

**Published:** 2014-03-17

**Authors:** F Sousa Cardosa, C Karvellas, N Kneteman, G Meeberg, P Fidalgo, S Bagshaw

**Affiliations:** 1 University of Alberta, Edmonton, Canada

## Introduction

Cirrhotic patients with Model for End-stage Liver Disease (MELD) score ≥40 have high risk of death without liver transplant (LT) [1]. This study aimed to evaluate these patients' outcomes after transplant.

## Methods

The retrospective cohort included 519 adult cirrhotic patients who underwent LT at one Canadian center between 2002 and 2012. Primary exposure was severity of end-stage liver disease measured by MELD score at transplant (≥40 vs. <40) [2]. The primary outcome was duration of first ICU stay after LT [3]. Secondary outcomes were duration of first hospital stay after LT, rate of ICU readmission, re-transplant rate, and survival rates.

## Results

On the day of LT, 5% (28/519) of patients had a MELD score ≥40. These patients had longer first ICU stay after LT (14 vs. 2 days; *P *> 0.001). MELD score ≥40 at transplant was independently associated with first ICU stay after transplant ≥10 days (OR, 3.21). These patients had longer first hospital stay after LT (45 vs. 18 days; *P *< 0.001); however, there was no significant difference in the rate of ICU readmission (18% vs. 22%; *P *= 0.58) or re-transplant rate (4% vs. 4%; *P *= 1.00). Cumulative survival at 1 month, 3 months, 1 year, 3 years, and 5 years was 98%, 96%, 90%, 79%, and 72%, respectively. There was no significant difference in cumulative survival stratified by MELD score ≥40 versus <40 at transplant (*P *= 0.59).

## Conclusion

Cirrhotic patients with MELD score ≥40 at transplant utilize greater postoperative health resources; however, they derive similar long-term survival benefit with LT.
